# Necessary additional steps in ultrasound guided central venous catheter placement: getting to the heart of the matter

**DOI:** 10.1186/s13054-017-1900-1

**Published:** 2017-12-19

**Authors:** Thei S. Steenvoorden, Jasper M. Smit, Mark E. Haaksma, Pieter R. Tuinman

**Affiliations:** Department of Intensive Care Medicine VUmc, Department of Intensive Care Medicine, Room ZH - 7B-90, De Boelelaan 1117, PO Box 7057, 1007MB Amsterdam, The Netherlands

**Keywords:** Intensive care, Central venous catheter, Ultrasound, Ultrasound guidance

With great interest we read the recent article on ultrasound (US)-guided central venous catheter (CVC) placement by Saugel et al. [1] and we completely agree with the authors on the importance of US guidance in CVC placement. However, we do believe that their protocol is incomplete. The authors’ recommendations rely solely on wire, needle, and CVC position confirmation in the entry vein, which does not provide the physician with information regarding correct CVC position or placement-associated complications.

First of all, the procedure’s success rate can be increased by adding guidewire confirmation in the right atrium. Research has shown that US confirmation of the guidewire’s position in the right atrium via the subcostal acoustic window is a reliable tool for assuring correct CVC placement. The guidewire should be moved into the right atrium until the tip can be visualized as a hyperechogenic line on the US image. The wire should then be pulled back until the tip is no longer visible through this acoustic window. Inserting the CVC after these steps results in a very reliable position [2, 3].

Secondly, even though it is beyond the scope of the research, we would like to emphasize that ultrasonography can also be used to accurately diagnose CVC misplacement and related complications such as pneumothorax. The CVC can unwantedly loop or migrate into various veins, for example, contra- or ipsilateral situated veins such as the subclavian or internal jugular veins [4]. Thus, in order to accurately determine malposition after the procedure, bilateral subclavian views should be integrated in the diagnostic US protocol as well. Furthermore, US signs, including “lung sliding”, “B-lines”, “seashore sign”, and the “lung point”, can help in the diagnosis of pneumothorax. Thereby, US imaging has been shown to be an even more accurate tool than chest X-ray in confirming this diagnosis [5].

A complete and thorough US protocol, including guidewire confirmation in the right atrium and post-procedural scanning for complications, will not only contribute to reducing periprocedural complications but will also make the still obligatory follow-up through chest X-ray obsolete, resulting in lower costs and less exposure to radiation for the patient.

## Abbreviations

CVC: central venous catheter
US: ultrasound
